# A Meta-Analysis of the Reliability of Second Language Listening Tests (1991–2022)

**DOI:** 10.3390/brainsci14080746

**Published:** 2024-07-25

**Authors:** Yuxin Shang, Vahid Aryadoust, Zhuohan Hou

**Affiliations:** 1 National Institute of Education, Nanyang Technological University, Singapore 639798, Singapore; NIE22.SY7766@e.ntu.edu.sg; 2 School of International Studies, Zhejiang University, Hangzhou 310058, China; h_zh@zju.edu.cn

**Keywords:** listening assessment, meta-analysis, moderator analysis, reliability generalization, validity arguments

## Abstract

To investigate the reliability of L2 listening tests and explore potential factors affecting the reliability, a reliability generalization (RG) meta-analysis was conducted in the present study. A total number of 122 alpha coefficients of L2 listening tests from 92 published articles were collected and submitted to a linear mixed effects RG analysis. The papers were coded based on a coding scheme consisting of 16 variables classified into three categories: study features, test features, and statistical results. The results showed an average reliability of 0.818 (95% CI: 0.803 to 0.833), with 40% of reliability estimates falling below the lower bound of CI. The presence of publication bias and heterogeneity was found in the reliability of L2 listening tests, indicating that low reliability coefficients were likely omitted from some published studies. In addition, two factors predicting the reliability of L2 listening tests were the number of items and test type (standardized and researcher- or teacher-designed tests). The study also found that reliability is not a moderator of the relationship between L2 listening scores and theoretically relevant constructs. Reliability induction was identified in reporting the reliability of L2 listening tests, too. Implications for researchers and teachers are discussed.

## 1. A Meta-Analysis of the Reliability of Second Language Listening Tests (1991–2022)

In language assessment, reliability is construed as the degree to which a test consistently and precisely gauges the same underlying construct over time, across test forms, and/or within a single test, ensuring dependable and trustworthy results [1] (pp. 30–32). A well-designed language test should demonstrate consistency in test scores under different conditions to accurately mirror test takers’ level of language knowledge and proficiency [2,3]. In contrast, an unreliable language test would yield random scores under different circumstances [4]. This lack of score reliability can increase the likelihood of committing type I errors and might undermine the accuracy of *p*-value and effect size estimation in multivariate analysis [5]. Reliability also forms a critical component of the “validity argument” or “assessment use argument (AUA)” framework, which is an evidence-based approach for justifying the interpretations and applications of test scores [6]. In validation research, high values of coefficient alpha have been perceived as evidence supporting the generalization inference and, consequently, the entire validity argument (e.g., [7,8]).

Four types of reliability analysis pervade the field of second language (L2) assessment: internal consistency of test items, parallel-forms reliability, test–retest reliability, and inter-rater reliability (see [5] for a review). Internal consistency refers to the togetherness of different test items tapping into the same construct in a language assessment. This type of reliability analysis is employed to examine the internal consistency of test items and the ability to differentiate between learners of varying skill levels [1]. Test–retest reliability represents another type of reliability, involving the consistency of test scores obtained by administering the same test to a group of individuals on two different occasions within a defined time frame and then calculating the correlation between the two sets of scores [5]. In addition, parallel-forms reliability is determined by correlating the scores procured on two distinct versions of the same test. High correlations indicate high parallel-forms reliability. Finally, interrater reliability assesses the consensus among different raters in their assessment decisions. Establishing interrater reliability is essential when different researchers adopt the same measurement on the same sample and compare the correlation of their different results. If the ratings are similar, the test has high interrater reliability [9]. Several methods exist for determining rater agreement, including percentage agreement, Cohen’s Kappa, Fleiss’ Kappa, and many-facet Rasch measurement, among others. Additionally, the intraclass correlation coefficient (ICC) is used to evaluate the consistency or agreement among measurements made by multiple raters evaluating the same construct on continuous or ordinal scales. These methods differ according to the number and type of raters, the measurement scale used, as well as the specific research context. In sum, different forms of reliability address distinct facets of a test’s consistency and precision.

In the context of listening assessment, researchers frequently utilize two forms of reliability measures: internal consistency reliability and inter-rater reliability (when listening is integrated with production skills like writing) [10]. In addition, Miao [10] found that researchers tend to prefer standardized listening tests in their studies when investigating research questions related to L2 listening, as they may deliver higher internal consistency compared to locally constructed tests. In a reliable listening test, the targeted listening skills are assessed with greater precision, thus maximizing the probability of consistent test scores across multiple administrations (see [11]). Internally, such listening tests would have a strong consistency, with all test items precisely measuring the same “attribute” [12] although the attribute they measure might not necessarily be the targeted construct. If the listening test involves subjective marking, such as in integrated tests, there should be a high level of agreement between different raters (inter-rater reliability), and if various versions of the test exist, they should all yield similar results (parallel-forms reliability) [9]. Nevertheless, as Fulcher and Davidson argued, in language assessment “it is very rare for [reliability] to be calculated in ways that actually involve giving multiple tests over time, or many similar tasks [1] (p. 30).” Thus, as a workaround, the estimation of internal consistency reliability through using the coefficient alpha has been commonly adopted in the field.

Despite the significance of reliability in language assessment, research on the reliability of L2 listening tests remains conspicuously sparse and, no study to our knowledge has systematically investigated the variables potentially impacting the internal consistency of these tests. To bridge this gap, this study uses a reliability generalization (RG) meta-analysis with four main objectives: (1) to synthesize the internal consistency of L2 listening tests; (2) to examine the heterogeneity and potential bias of the L2 listening assessment publications; (3) to investigate factors statistically associated with the variability of reliability estimates; and (4) to research the impact of reliability on the relationship between listening test scores and other constructs.

RG is a method for combining and analyzing reliability estimates from various studies to identify factors that affect the consistency of those estimates [13] (p. 5455). The application of RG meta-analysis, as suggested by Henson and Thompson [14], can help researchers conduct future studies, predict the reliability of measurements, and make informed decisions regarding research approaches. This method will be elaborated on in the following sections.

## 2. Literature Review

### 2.1. L2 Listening Assessment

Listening is a combinatorial multifaceted process that requires listeners to recognize the acoustic signal, comprehend details and implied information, and interpret speakers’ intentions and attitudes (see [15,16,17,18,19] for different perspectives on listening). This is accomplished through a broad array of linguistic knowledge, encompassing phonology, lexis, syntax, semantics, and pragmatics, as well as non-linguistic skills such as understanding the topic, context, and general world knowledge [16]. Additionally, online processing adds further complexity to listening, as listeners engage in real-time interpretation and understanding of the spoken language. This simultaneous information processing can make listening a challenging skill, as noted by scholars like Buck [17], Goh and Vandergrift [20], Field [21], and Rost [22]. Due to the latency and lack of observable products unlike writing and speaking, assessing listening is technically more complicated and time-consuming than assessing other language skills.

Previous studies have explored different facets of the L2 listening construct as assessed by listening tests [16,19,23,24,25,26,27,28,29,30,31,32,33,34]. Aryadoust and Luo [16] identified three distinct approaches to defining and operationalizing L2 listening constructs: subskill-based, process-based, and attribute-based.

The subskill-based approach posits that listening is not a singular skill but rather involves various interrelated mental abilities that contribute to the process of listening comprehension [22,23,35]. Advocates of this atomistic approach argue that listeners utilize and integrate various subskills—such as knowledge of the sound system, comprehension of local, global, and inferred meanings, and communicative listening skills—to attain listening comprehension, a concept that has been extensively explored in the literature [16,17,21,23]. In line with this approach, previous studies have identified numerous L2 listening subskills [16]. Aryadoust and Luo’s [16] framework of L2 listening constructs, for example, recognizes 5 main-layer listening subskills along with 19 secondary subskills. This model includes linguistic knowledge, such as phonological, grammatical, semantic, and pragmatic understanding of L2 listening, which allows L2 learners to identify syllables and phonemes, decode words, and infer meanings [36]. These aspects of linguistic knowledge are fundamental in a listening model [37]. Using the subskill-based approach as a foundation, L2 listening test developers can examine L2 learners’ listening abilities and distinguish listeners’ comprehension for diagnostic purposes [16].

The process-based approach to L2 listening is featured by three key aspects: affective, behavioral, and cognitive processes [38]. Affective processes pertain to listeners’ emotions and feelings, whereas behavioral processes involve the actions of listeners, such as eye contact and body language. Cognitive processes involve internal and neurocognitive processes that allow listeners to understand explicit and implicit information in the auditory input. The process-based approach is less frequently adopted in L2 listening test development, even though theoretical discourse within listening research tends to emphasize the role of cognitive process over affective and behavioral processes [16]. This may be attributed to a less comprehensive understanding of listening processes in previous research [39] and the scope and intricacies of cognitive processes in real-life listening [16]. An additional factor contributing to this shortfall is the difficulty in creating test items that effectively target distinct cognitive processes in listening. This challenge arises because these processes are often closely intertwined [21]. Designing test items that isolate and measure separate cognitive functions requires careful consideration due to their interconnectedness [40]. Furthermore, administering such tests in common L2 testing environments may require access to specialized equipment, which is often impractical for many language classrooms.

The attribute-based approach to L2 listening highlights the influence of various factors on listening assessment outcomes, spanning from text and test characteristics to learners’ non-cognitive processes [41] (p. 121). In Aryadoust and Luo’s [16] framework of attribute-based approach to L2 listening constructs, there are three primary attributes: task-related attributes, listener-related attributes, and others. The task-related attributes consist of visual content, test features, and features of aural input, which can significantly contribute to the authenticity of an L2 listening assessment. Previous studies have investigated the impact of listener-related attributes, such as participants’ demographics, on listening performance [42]. However, the effect of test features, including test items and task features, on the fairness of assessment requires further research [16]. Other dimensions of attributes include testlet effects and input level factors. A testlet is a packet of test items that are administered together. Research shows that neglecting testlet effects can result in an overestimation of test reliability [43,44] and biased test parameter estimations [45]. The input level, referring to the modality effect in listening tests, influences listening comprehension test performance in multiple ways [16,46]. For example, Liu and Aryadoust [46] have shown that video input positively enhances L2 listening test performance.

In summary, the subskill-based, process-based, and attribute-based approaches to L2 listening comprehension provide useful insights into the various types and layers of L2 listening constructs. Numerous studies have been conducted to measure constructs of L2 listening comprehension from these perspectives. However, the trustworthiness and reliability of L2 listening assessments have been overlooked by various researchers [10,16]. It is important to explore the reliability of L2 listening tests and the potential factors influencing reliability, and therefore an investigation would help enhance the replicability of measurements and identify factors that affect replicability [9] in L2 listening assessments.

### 2.2. Factors Affecting Reliability in Language Assessment

Reliability is of paramount importance for an accurate evaluation of an individual’s language and listening ability. Researchers have investigated multiple factors that may influence the reliability of language test scores, including test type, test length or number of items, item difficulty and item discrimination, item type, interaction between test takers and tests, and scoring of the test. These factors are fundamentally concerned with the attribute-based approach to L2 listening assessment, which delineates the diverse attributes that can impact L2 listening test performance [16]. However, while separate investigations have been carried out on the reliability and attributes of L2 listening tests, a direct examination of the nexus between these attributes and reliability has largely been overlooked. This is an important oversight, as examining this relationship could help to bridge the gap between construct definition and operationalization and reliability. In the validity argument framework [7], while reliability is viewed as a component of validity, its potential modulation by the attributes constituting the L2 listening construct, as conceptualized within the attribute-based approach, remains nebulous. The following section discusses test attributes that affect reliability.

*Test type.* Listening assessments generally fall into two categories: standardized tests and researcher-designed or teacher-designed tests. The choice between standardized tests and tailor-made assessments by researchers or teachers in listening comprehension studies hinges on the specific objectives and contexts of the research. While standardized tests offer a uniform measure of listening ability, custom-designed tests may allow for greater flexibility and specificity, thus aligning with the unique requirements of each study. Studies showed that standardized tests yield scores with higher reliability than teacher-designed English listening or language tests (e.g., [9,10,47,48]). As standardized listening tests are designed to differentiate varying levels of test takers, this factor could increase their reliability. In contrast, listening tests, typically developed by teachers, are more prone to producing “inconsistent” results than standardized tests [47], partly due to the limited number of test items or tasks included.

*Test Length and Number of Items.* The length of a test specifically referring to the length of the listening passage is considered a major factor that affects the reliability of tests. Previous studies have shown that longer tests generally yield higher score reliability coefficients [47,49]. Similarly, research indicates that the duration of listening test materials affects the reliability of scores obtained by children [49]. Additionally, the breadth or scope of the test is a factor that can influence the reliability of these scores [47].

The number or quantity of test items in language assessments is also related to reliability. Specifically, the mathematical notation of coefficient alpha indicates that, all else being equal, coefficient alpha increases as the number of items in the test increases [50]. This notion has garnered empirical support, with research demonstrating that the internal reliability of tests increases by incorporating more on-target test questions or test items [1]. On the other hand, Livingston et al. [48] observed that the reliability of a multiple-choice test became more stable when the number of items exceeded 100. This might suggest that when the quantity of on-target items is already ample, it becomes challenging to further improve reliability by adding more items. Additionally, variations in the number of listening passages included in a test may yield different reliability outcomes, which can be attributed to the diversity and distribution of content. Further research is needed to explore optimal passage lengths and numbers.

*Item Difficulty and Discrimination.* Item difficulty and item discrimination can significantly impact the reliability of language assessments [9]. Item difficulty, measured on a scale from 0 to 1, is the degree to which students choose the correct answer on the test, with lower values indicating more difficult test items and higher values easier ones. In item response theory (IRT), maximum likelihood estimation or related methods are commonly employed for estimating item difficulty and discrimination parameters [51]. (Maximum likelihood estimation (MLE) in IRT is a statistical method used to estimate the properties of test items, such as their difficulty and discrimination. MLE works by finding values for these parameters that maximize the probability of the observed responses given by test-takers. In other words, it is a way to determine how challenging an item is and how well it differentiates between test-takers of different ability levels, based on how test-takers with varying abilities have responded to the items (see [51]).). Several studies suggested a strong link between item difficulty and test score reliability. Al-zboon et al. [52] argued that the degree of difficulty of multiple-choice test items has a significant bearing on the reliability of test scores, with optimal reliability coefficients emerging from items of medium difficulty. According to Fulcher and Davidson [1], test items with a difficulty value of 0.5 could maximize the reliability of language assessment. They also argued that uniform item difficulty produces more reliable scores, though it might risk the validity by potentially excluding items measuring unique construct facets.

Item discrimination, referring to the degree to which test items effectively discriminate between students of different levels, also affects reliability [53]. In classical test theory, the point-biserial correlation, which measures the relationship between item scores and total test scores, is employed to assess item discrimination. The index ranges between −1 and 1, with negative correlations indicating that students with low proficiency score higher than high-proficiency students on the test [54]. Another method, the discrimination index, a relatively simple, non-parametric method, quantifies how well an item discriminates between higher and lower-scoring groups. English listening tests that effectively distinguish between low-proficiency and high-proficiency students tend to produce more reliable scores [47]. Furthermore, a substantial dispersion in test scores often leads to heightened reliability [9], whereas when test score distribution is uniform, the reliability of language tests tends to experience a downturn [1] (p. 106).

*Item Type.* The poor choice of item type is another factor leading to unreliable language test scores [55]. There are two major item types in language tests: selected-response items, such as multiple-choice questions (MCQs), and constructed-response items, like open-ended questions. Selected-response items, especially MCQs, are commonly adopted in listening assessments to examine students’ listening comprehension. By contrast, constructed-response items in listening tests require learners to answer questions using their vocabulary knowledge and writing skills (see [56]). Research indicates that employing the MCQ format in language assessments can lead to higher test reliability [48]. Many language tests exclusively use the MCQ format because they are more likely to yield higher reliability [55]. However, it has been argued that this kind of test method lacks authenticity in assessing language ability [57], largely because using MCQs is hardly representative of communication in any target language use domain. Therefore, integrated tests, including at least two language skills, are employed increasingly in English language assessments, although the reliability of such integrated tests still requires further research [55].

### 2.3. Reliability Generalization Meta-Analysis of L2 Research

Reliability generalization (RG) is a meta-analytic approach used to examine average reliability estimates and understand the variability associated with them [58]. RG is an effective method to correct misinterpretations and misunderstandings of the concept of reliability, a concept often inadequately addressed or overlooked [59]. Researchers have widely adopted RG meta-analysis in various fields to investigate the reliability of instruments or measurements such as in the medical field [60] and psychology [61]. However, the RG meta-analysis has not received much attention in the field of L2 research and only a few studies have been conducted on RG meta-analysis of L2 research [4,62,63].

Watanabe and Koyama [62] investigated L2 cloze tests in their internal consistency using the RG meta-analysis method, providing an application of RG meta-analysis in L2 research. The results showed that internal consistency, as assessed by various statistical indices including coefficient alpha, Kuder–Richardson Formula 20 (K-R20), and Kuder–Richardson Formula 21 (K-R21), varied considerably, with studies employing K-R20 demonstrating the highest reliability index in L2 cloze tests. Furthermore, Watanabe and Koyama [62] found that both the scoring methods and the parts of passages removed significantly affect the reliability of L2 cloze tests. They found that L2 cloze tests with acceptable scoring, involving deletion of the seventh word and rational deletion, yield the highest reliability.

Plonsky and Derrick [4] provided an overview of reliability in L2 research to assist researchers in understanding reliability estimates and examining factors that moderate instrument reliability (such as study and instrument features). In terms of study features, there was no relationship between sample size and reliability. However, Plonsky and Derrick [4] found that reliability tends to increase when there is less variation in the responses or conditions. This suggests that, in line with Fulcher and Davidson [1], instrument reliability might decline for L2 learners with low language proficiency, or among cohorts of uniformly high-ability learners, due to reduced dispersion. Moreover, instrument reliability was found to be higher in lab-based studies than in classroom-based studies. When examining instrument features, the number of items emerged as a major moderator of reliability, which is consistent with other RG meta-analysis studies (e.g., [63]). Additionally, the reliability of item types with a large range of responses was found to be higher than those with a small range of responses like MCQs. They also emphasized the significance of selecting the appropriate reliability coefficient index and suggested that researchers proffer a thorough interpretation of their chosen index.

In addition, Aryadoust et al. [63] investigated the RG of language learning motivation (LLM) in L2 research. The study identified heterogeneity and potential publication bias in the reliability coefficients of LLM tools, revealing considerable variation in the reliability statistics reported by researchers. It also indicated that LLM researchers were more likely to report high reliability coefficients than low ones. Furthermore, the study found that the number of items in LLM tools affects internal consistency, with longer questionnaires generally yielding higher reliability coefficients [63].

### 2.4. Effect of Reliability on the Relationship between Listening and Other Constructs

While reliability is viewed as a prerequisite for higher-order validity inferences such as extrapolation or criterion validity [7], this postulated relationship has rarely been subjected to scientific scrutiny specifically within the context of listening tests. Most previous studies shed light on the relationship between linguistic as well as non-linguistic factors and listening comprehension but failed to explore potential variables affecting or moderating the relationship.

Zhang and Zhang [64] conducted a meta-analysis to investigate the connection between vocabulary knowledge and L2 listening comprehension. The results indicated that meaning recall knowledge, word association knowledge, and morphological knowledge significantly correlated with L2 listening comprehension. Among these constructs, meaning recall knowledge showed the strongest correlation. Listening strategy instruction also boosts L2 listening comprehension [65]. According to Dalman and Plonksy [65], the number and type of strategy instruction can affect the effectiveness of listening strategy training on L2 listening. Additionally, In’nami et al. [27] explored the relationship between metacognitive factors and L2 learners’ listening comprehension, finding that metacognitive awareness and working memory significantly contributed to L2 listening performance. This may also provide evidence for the frameworks that postulate listening is dependent upon cognitive abilities [17,66]. Given that reliability underpins higher-order validity inferences, it is expected that the reliability of listening test scores will account for some of the variance in the aforementioned relationships. For instance, the correlation between listening test scores and external measures like vocabulary and grammar can be interpreted as evidence supporting the explanation inference. This evidence is partly predicated on reliability, which constitutes the conclusion or claim of the generalization inference in a given validity argument [7,67]. However, this assumption has consistently been taken for granted and has never been submitted to any rigorous evaluation.

## 3. The Present Study

As previously discussed, further research is needed on the reliability of second language (L2) listening tests, including the factors affecting this reliability, and the relationship between test score reliability and other factors in L2 listening assessments. While the former analysis can demonstrate what factors influence L2 listening test scores’ reliability, the latter can reveal how reliability can predict or influence the relationship between listening test scores and other relevant measures. The present study seeks to address this gap by conducting a meta-regression and moderator analysis within the framework of reliability generalization (RG) [68], respectively, to explore possible variables that influence the internal consistency of L2 listening tests and the nexus between reliability and other linguistic measures associated with L2 listening assessments.

Reliability induction is a key concept in reporting reliability, where researchers cite reliability coefficients from prior studies to support the reliability of their current research data [69]. Reliability induction can be problematic because the sample characteristics may differ between the two studies [70]. Since reliability induction is reported in several L2 listening studies, it is desirable to investigate its impact on the reliability of L2 listening tests.

The following are the research questions (RQs) of the study:

RQ1: What is the average degree of reliability of L2 listening tests in the published literature?
RQ2: What variables affect the reliability of L2 listening tests?
RQ3: How is the relationship between L2 listening test scores and constructs moderated by the reliability coefficients of L2 listening tests?
RQ4: To what extent does the field of L2 listening assessment rely on reliability induction?

## 4. Method

### 4.1. Dataset

In this study, Scopus [71], one of the largest online databases available, was chosen as the main database since it contains more studies about L2 listening tests. The Web of Science (WOS) [72] was also used to cross-check the results from Scopus and ensure the inclusion of a comprehensive dataset.

The relevant publications were searched in Scopus and WOS using keywords as follows: “L2 listening tests”, “listening comprehension”, “second language listening”, and “listening”. A search query (see Supplemental Material S1) which contains 55 journals was employed in the search process in Scopus. Given that the study concentrated on second language listening tests, journals related to (second) language teaching, language learning, and language assessment within the field of applied linguistics were chosen from Scopus. All papers selected in the study were published journal articles, while reviews and editorials (*k* = 10), books (*k* = 4), conference presentations (*k* = 8), and book chapters (*k* = 21) were not included in the study, as they did not present any specific studies with reliability statistics. Although reliability coefficients were reported in three book chapters, including them alongside a substantial number of journal articles could skew the sample towards articles, due to their significantly larger representation. Consequently, we decided to exclude these book chapters from the data. In addition, only quantitative studies were chosen in the study, as they provided the reliability coefficients of L2 listening tests. There were no restrictions on the publication year of the studies, as the current research aimed to analyze the reliability of L2 listening tests using extensive data. A set of inclusion and exclusion criteria was applied to minimize potential bias in the data selection process (see Table 1).

**Figure 1:**
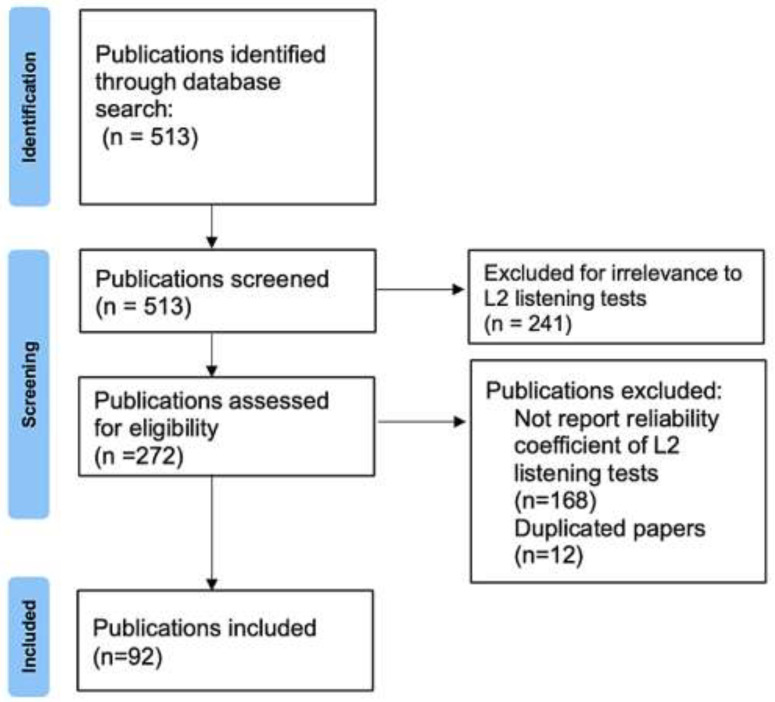
PRISMA of the study.

As shown in Figure 1, we applied the preferred reporting items for systematic reviews and meta-analysis (PRISMA), which demonstrates the searching, screening, and selection process. After searching the keywords on Scopus and WOS, 513 papers were found in total. Next, the abstracts of the papers were screened and reviewed. The study excluded a total of 241 journal articles unrelated to L2 listening assessment and 168 studies that did not report the reliability of L2 listening tests. In addition, 12 duplicated papers were also excluded from the study. There were three types of primary studies reporting the reliability estimates: studies employing a single listening test with a single sample of test takers (*k* = 69); studies employing a single listening test with multiple samples of test takers (*k* = 3); and studies conducting multiple listening tests with a single sample of test takers (*k* = 20). Overall, a total number of 92 papers published between 1991 and 2022 in 28 journals yielding 122 reliability estimates in L2 listening tests were included in the study (see Supplemental Materials S2 and S3).

### 4.2. The Coding Scheme

A coding scheme adapted from Plonsky and Derrick [4] was used to select and organize the study characteristics and reliability estimates from the papers. The scheme included sixteen variables, categorized into study description, test features, and statistical results (see Table 2).

To ensure coding reliability, inter-coder reliability was assessed using the simple agreement percentage. Another experienced coder re-coded the data after taking the training protocol outlined by Plonsky and Oswald [73]. Except for background and item type, where the agreement was 96% (i.e., the raters disagreed only on 4 to 5 cases in each variable), a perfect agreement (100%) was observed in the coding of all the rest of the categories. For the disagreement cases, we revised the categories and confirmed them with the second coder to ensure reliability.

### 4.3. Meta-Analytic Procedures

Reliability coefficients for L2 listening test scores were collected. All included papers reported the Cronbach’s alpha coefficient for the L2 listening tests used. To address RQ 1, descriptive statistics summarizing the reliability coefficients were calculated using *SPSS* (version 25). The *Metafor* package [74] in *RStudio* (version 4.2.1) [75] was also used to assess the average reliability, heterogeneity, and publication bias for the first research question (R code provided in Supplemental Material S4). Heterogeneity refers to the variability or differences in the data. Significant heterogeneity suggests that the effect size, and in this case reliability coefficients, may vary across studies due to several factors such as the sample size, study contexts, and so on [68]. Several statistics were calculated to evaluate data heterogeneity, including tau-squared (*τ*^2^, ranging from 0 to any positive value), the *I*^2^ statistic (ranging from 0% to 100%), and the *Q*-test [76,77,78]. To assess publication bias, a funnel plot [79] and Egger’s regression test [80] were used. To address the potential dependency issue in our dataset, we applied a linear mixed-effect model for the average reliability estimate.

To address the second research question, a meta-regression analysis was performed to explore potential factors that might impact the reliability coefficient. Informed by Plonsky and Derrick [4], we conducted a linear regression analysis on the study features, test features, and statistical results to find out the significant predictors of reliability coefficients. Specifically, we examined the number of items, test type, item type, participants’ background, gender, and sample size across primary studies to identify the predictors that may influence the reliability coefficients of L2 listening tests. Due to a significant amount of missing data in the dataset (please refer to the enclosed dataset), other variables were excluded from the analysis. This lack of data arises from previous studies not offering detailed information on L2 listening tests. In the meta-regression analysis, *R*^2^, adjusted *R^2^*, and regression coefficients were estimated and interpreted.

To address research question 3, we reviewed journal articles collected in the meta-analysis and classified them according to their topics. Next, the studies that examined the relationship between or the effect of certain variables on L2 listening comprehension were selected and identified to perform a non-parametric Spearman’s rank correlation analysis. This correlation assesses the ordinal association between two variables and Spearman’s rank correlation is commonly used in this analysis due to its ability to effectively handle non-linear relationships and rank-order differences [81]. A total number of 17 published articles examining the relationship of relevant constructs including five papers on strategy instruction as well as metacognition and L2 listening, and 12 research papers on the relationship of vocabulary and L2 listening comprehension were selected in the study to determine whether the nexus between L2 listening test scores and other constructs is moderated by the reliability coefficients of the L2 listening tests.

Finally, to address research question 4, a Mann–Whitney U test was used to examine differences between the median reliability of induction studies and other L2 listening test studies. The Mann–Whitney U test, a non-parametric method, is employed when sample sizes are small or when the data do not meet the normality assumption required for parametric tests such as the *t*-test [82].

## 5. Results

### 5.1. Descriptive Statistics

Table 3 and Table 4 display the participants’ demographics and the contexts of the primary studies included in the meta-analysis. The tables also display the mean reliability statistics and associated standard deviations of the L2 listening tests, categorized according to the demographics of the test takers (Table 3) and study contexts (Table 4). In terms of participant demographics, most of the participants were university students (*k* = 68, 55.70%). Regarding gender distribution demonstrated in Table 3, only a minority of the studies exclusively involved female participants (*k* = 9, 7.30%), whereas the majority included both male and female participants (*k* = 113, 92.70%). The majority of participants in the research studies were comprised of individuals with different first languages (*k* = 38, 31.10%) and speakers of Chinese (*k* = 37, 30.3%). Additionally, the target language in the primary studies was English in all the studies (*k* = 122, 100%). Table 3 further reveals that listening tests used to assess university students and EFL/ESL learners exhibit higher reliability (0.82 and 0.83, respectively), while tests used to measure the listening skills of cram school students show the lowest reliability (0.72). Gender-wise, mixed-gender groups have a marginally higher reliability (0.81) compared to female-only groups (0.80). Among L1 language speakers, studies of Arabic and Vietnamese speakers reported the highest reliability (0.86), while studies of Farsi speakers have the lowest (0.74). Variability in reliability is most pronounced among Japanese speakers (SD = 0.114), and the data also indicate that tests used to evaluate the listening skills of multilingual test takers have slightly lower reliability (0.79) compared to the overall mean for L2 English tests (0.81).

With regard to the study context in Table 4, the majority of the studies were conducted in China (*k* = 37, 30.30%), with Japan (*k* = 18, 14.80%) and Korea (*k* = 18, 14.80%) being the next highly researched contexts. In addition, 39 studies (32%) primarily utilized sample sizes ranging from 51 to 100 participants in the research of L2 listening tests. Among the countries, Australia and New Zealand had the highest mean reliabilities for L2 listening tests (0.87 and 0.89, respectively), with New Zealand also showing the least variability. Conversely, Iran and the US were at the lower end of reliability (0.78 and 0.76). When looking at sample sizes, larger studies with over 300 participants had lower reliability (0.78), while those with 201–250 participants achieved the highest reliability (0.89). Interestingly, studies with 51–100 participants and unreported sample sizes showed comparable mean reliabilities (0.79 and 0.81).

A summary of the features of L2 listening tests is presented in Table 5. Researchers tended to adopt standardized tests (*k* = 65, 53.20%) in their L2 listening studies. As demonstrated in the second column from the right side, the mean reliability of standardized tests (0.82) is higher than the mean reliability of researcher-designed tests (0.79). In addition, fill-in-the-blank items (*k* = 54, 44.30%) and multiple-choice questions (*k* = 53, 43.40%) as L2 listening item types were dominantly used as L2 listening test item types in the majority of the studies. The mean reliability of tests comprising both multiple-choice questions and fill-in-the-blank items was the highest (0.83), compared to fill-in-the-blank items (0.81) and multiple-choice questions (0.80). Additionally, many studies did not provide information on test setting (*k* = 81, 66.40%) or test form (*k* = 92, 75.40%).

Finally, Table 6 shows that the average number of items is 40.93, with a broad range spanning from 8 to 216 items. The substantial standard deviation (SD = 31.996) suggests a significant variation from the mean in the number of items.

### 5.2. Reliability Generalization Results

**Figure 2:**
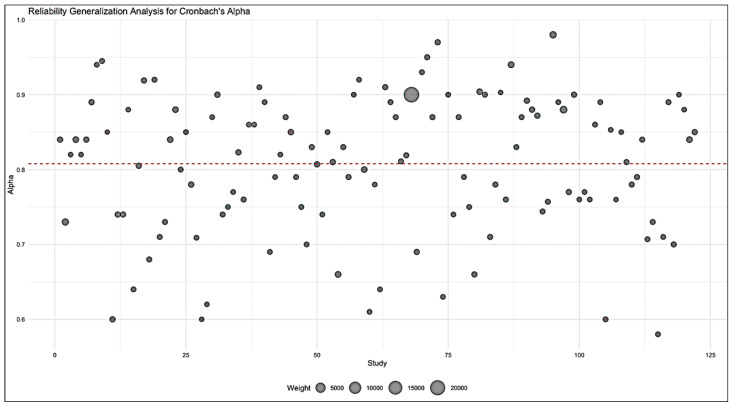
Bubble plot of reliability generalization analysis.

The analysis included 122 reliability coefficients from 92 papers on L2 listening tests. The reliability estimates of previous listening tests demonstrated dependencies primarily due to the involvement of the same participants in multiple tests. This repeated participation led to intrapersonal correlations in the data. To appropriately address this source of dependency, we applied a linear mixed-effect model. This model incorporated random effects for participants, effectively capturing the individual variations and dependencies arising from their multiple involvements. Through this approach, we could more accurately examine the reliability of the listening tests by accounting for and isolating the influence of study-specific factors on the overall data structure. Figure 2 demonstrates a bubble plot with the study organized horizontally, and the dashed horizontal line indicating the average reliability estimate. The observed Cronbach’s alphas ranged from 0.58 to 0.98 and the estimated average Cronbach’s alpha based on the random-effects model was *μ* = 0.818 (95% CI: 0.803 to 0.833), which is considered to be a high reliability coefficient. The 95% confidence interval provides a range of values within which we can be 95% confident that the true population reliability lies. The range from 0.803 to 0.833 indicates that the true reliability generalization coefficient for this sample of listening tests tends to fall in this range with a 95% level of confidence.

**Figure 3:**
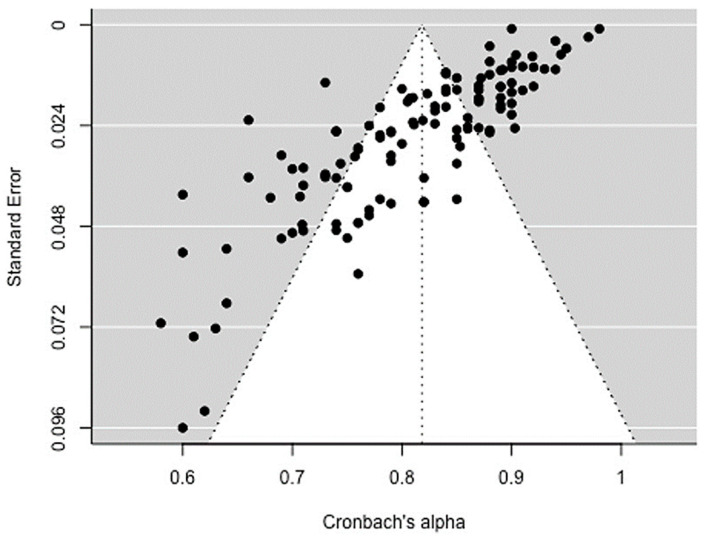
Funnel plot of the Cronbach’s alpha values.

Next, we examined potential publication bias in the dataset. Figure 3 presents a funnel plot that illustrates the relationship between Cronbach’s alphas and standard error measures. The plot provides preliminary insights into the presence of publication bias and exhibits an asymmetrical distribution of dots which appear rather distorted, with an uneven distribution of dots. The Egger’s regression test also revealed funnel plot asymmetry (*p* < 0.0001). This suggested the potential presence of bias and was followed up with statistical tests suitable for detecting heterogeneity.

The heterogeneity of the Cronbach’s alpha coefficients was first examined using the *Q*-test. The *Q*-test indicated significant heterogeneity among Cronbach’s alpha coefficients (*Q*(121) = 6704.24, *p* < 0.0001), suggesting that the variations in reliability estimates across studies are unlikely to be attributed solely to random variability or sampling error. In addition, the estimated between-study variance component represented as *τ*^2^ was found to be 0.006, suggesting a certain level of heterogeneity. The *I*^2^ statistic, representing the proportion of total variation attributable to heterogeneity, was calculated to be 98.95%, suggesting that a substantial portion of the observed variability in reliability coefficients across the studies is due to true differences rather than sampling error. Overall, these results reveal a substantial level of heterogeneity among Cronbach’s alpha coefficients, emphasizing the need for further investigation into the factors contributing to the observed differences. Thus, the next step comprised a meta-regression analysis to explore the potential factors causing the observed heterogeneity in Cronbach’s alpha values.

### 5.3. Meta-Regression Results

A meta-regression analysis was performed to identify potential causes of variance in Cronbach’s alpha coefficients. The R^2^ index was 0.26, indicating that 26.40% of the variance in the outcome variable was explained by the predictors. In addition, the adjusted *R*^2^, which adjusts the *R*^2^ for the number of predictors in the model, was 17.40, suggesting that, after adjusting, 17.4% of the variance in the outcome variable can be explained by the predictor variables, considering the model complexity and sample size.

The results of the ANOVA test of the meta-regression analysis are also examined. The sum of squares (SS = 0.261) measures the amount of variability explained by the regression model, indicating that the regression model explains 26.10% of variability in the outcome variable. The regression model provides a better fit to the data compared to the null model, as indicated by the higher F value, which suggests a stronger fit relative to the null model (*F*(13, 107) = 2.946, *p* = 0.001). Six variables were entered as independent variables in the meta-regression model: the number of items, test type, item type, background, sample size, and gender. As demonstrated in Table 7, among them, two variables, the number of items (continuous) and test type (categorical), were significant predictors in the study (*p* < 0.05). For the test type, there were two categories: standardized tests and researcher-designed tests. According to the results of the test type (*p* = 0.021), standardized L2 listening tests like IELTS, TOEFL, and other official tests tend to yield reliability coefficients that are higher than researcher-designed tests when used for research purposes. The rest of the variables and their levels were not statistically significant predictors of the reliability coefficients of L2 listening tests (*p* > 0.05). For the categorical variables such as item types and participant backgrounds, each category’s data are compared against a designated anchor or baseline category in the data. However, it should be noted that the baseline categories are not specified in the provided table. For item type, the baseline category is the constructed-response item type, and for background, the baseline category is identified as adult learners.

### 5.4. Meta-Correlation Analysis

A total of 17 articles investigated the effect of variables such as vocabulary, metacognition, working memory, and listening strategy on L2 listening. A total of 14 studies out of these 17 studies used standardized tests of L2 listening tests. Table 8 presents the descriptive statistics of these studies. Among the studies, researchers mostly investigated the effect of vocabulary on L2 listening (*k* = 12, 9.80%), while only one study investigated the effect of strategy instruction. The mean correlation of working memory and L2 listening tests was the highest (M = 0.62) compared to other studies. The mean reliability coefficients of L2 listening tests across different correlational studies are also presented in Table 8. The mean of the reliability of studies reporting a relationship between metacognitive awareness and listening test scores is the highest (M = 0.87), while the reliability of the single study reporting the correlation between strategy instruction and listening test scores is the lowest (M = 0.80).

Next, due to the small size of the data, a nonparametric meta-correlation analysis of the relationship and internal consistency was conducted to explore whether there was a relationship between correlation coefficients and reliability coefficients of L2 listening tests. The results of the correlation analysis suggested that correlation coefficients do not correlate with reliability coefficients (*r* = 0.328, *p* = 0.199) at the significance level of 0.05. Additionally, correlation analysis between the correlation coefficients of vocabulary and L2 listening comprehension and the reliability coefficients of L2 listening tests was performed. The results showed no relationship between the correlation coefficients of vocabulary and L2 listening comprehension and the reliability coefficients of L2 listening tests (*r* = 0.098, *p* = 0.761). Thus, the association between vocabulary and L2 listening comprehension does not appear to be related to the reliability of L2 listening tests. The analysis did not include a correlation analysis of the other three categories (metacognitive awareness, working memory, and strategy instruction) because of their relatively small sample sizes.

### 5.5. Reliability Induction Analysis

Table 9 shows descriptive statistics for studies employing reliability induction for determining the reliability of listening instruments, alongside those that directly calculated reliability. Most studies actually calculated the reliability of L2 listening tests, with 117 studies, accounting for 95.90% of the total, while a smaller proportion, consisting of 5 studies, or 4.10%, reported reliability coefficients derived from prior research.

**Figure 4:**
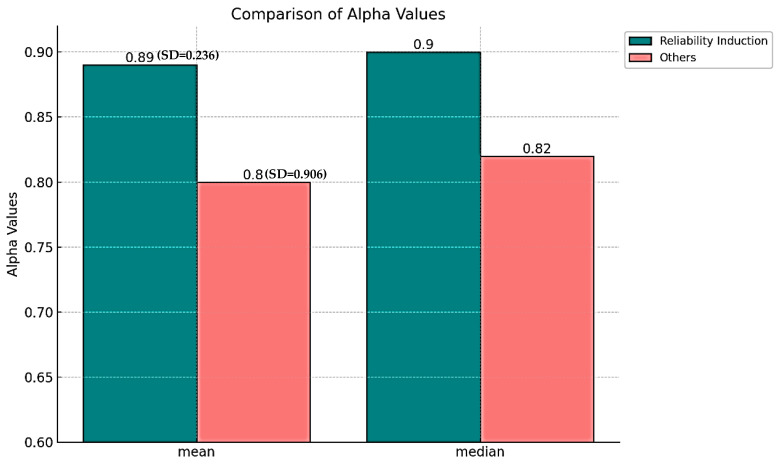
Comparison between the mean and median of reliability coefficients of reliability induction and other studies.

Relatedly, Figure 4 presents the comparison of the mean and median of reliability coefficients between L2 listening studies reporting reliability induction and other studies. The mean and median of reliability coefficients of reliability induction (M = 0.89, Mdn = 0.90, SD = 0.263) were higher than the mean and median of other studies (M = 0.80, Mdn = 0.82, SD = 0.906). In addition, a Mann–Whitney U test was conducted to investigate the impact of reliability induction on the reliability of L2 listening tests. The result showed that reliability induction significantly influences the reliability of L2 listening tests (*z* = −2.604, *r* = 0.235, *p* = 0.009).

## 6. Discussion

In this study, we conducted a reliability generalization (RG) meta-analysis to determine the average reliability of L2 listening tests from previous research. We also examined heterogeneity and publication bias to explore the transparency, robustness, and validity of the meta-analysis. A total of 122 reliability coefficients from L2 listening tests, derived from 92 published journal articles, were collected and analyzed. The findings are discussed in the following sections.

### 6.1. Research Question 1: Reliability Generalization of L2 Listening Tests

#### 6.1.1. Reliability of L2 Listening Assessments

The study revealed that the average reliability coefficient of L2 listening tests was 0.818 (95% CI: 0.803 to 0.834) in the meta-analysis, reflecting a great internal consistency of L2 listening assessments on average—as publication bias was detected in the meta-analysis, a point that will be discussed later, this result should be approached with caution. According to Taber [83], Cronbach’s alpha above 0.70 is preferable, and in the field of education, a value above 0.80 is considered both acceptable and highly reliable. Dalman and Plonsky [65] observed high reliability in instruments within the domain of L2 listening, noting that internal consistency was the most commonly employed method in these studies. The relatively high aggregated reliability of L2 listening test scores indicates that L2 listening tests may yield consistent and dependent scores across various studies. This, in turn, enables educators to precisely evaluate students’ L2 listening proficiency with a commendable level of internal consistency within the listening test. The use of a reliable L2 listening test ensures a more accurate representation of test takers’ true listening proficiency by reducing the impact of measurement errors and external factors. However, it is important to note that 49 out of the 122 reliability coefficients were below the lower boundary of the 95% confidence interval, which indicates that approximately 40% of the reliability coefficients are not consistent with the expected range of reliability coefficients for the given level of confidence.

Notably, the range of reliability estimates displays considerable variability, as reliability coefficients (Cronbach’s alpha) below 0.70 are regarded as indicating insufficient internal consistency for an assessment instrument, as highlighted by Taber [83]. Within the context of the RG meta-analysis focusing on L2 listening tests, the highest reliability coefficient recorded was 0.98, while the lowest was 0.58. Despite the overall favorable average reliability of L2 listening tests, it is important to acknowledge the existence of several reliability coefficients in L2 listening research that remained at lower levels. Instances of poor reliability within L2 listening tests imply that the outcomes of L2 listening comprehension within research may lack reliability and generalization power for the broader population. The finding is also consistent with Plonsky and Derrick’s [4] study that showed instrument reliability of L2 listening is relatively low compared to L2 reading, writing, and speaking. Consequently, it becomes imperative for researchers to prioritize the consideration of reliability when selecting or designing L2 listening tests for their studies.

#### 6.1.2. Publication Bias

Publication bias was also found in the RG meta-analysis of L2 listening tests. This finding suggests that researchers might be inclined to selectively report higher reliability coefficients for their L2 listening test studies, possibly omitting or underreporting lower reliability coefficients. Thornton and Lee [84] provided insights into various reasons underlying publication bias in single studies. One primary cause is the design and analysis of single studies, encompassing factors like study context and researchers’ expectations. Studies with small sample sizes, for instance, may yield insufficient or insignificant statistical results, thereby contributing to publication bias. Additionally, certain researchers might hold preconceived notions about their expected outcomes before analyzing the data, potentially influencing the reporting of statistical results. Furthermore, publication bias can also be attributed to the perspectives of journal editors and reviewers. They may favor articles with positive results over those with negative outcomes. “Negative” studies or studies with low-reliability coefficients often face heightened scrutiny and a higher likelihood of rejection due to perceived lower quality, even if some of these studies can be well-designed [84]. It is crucial for researchers, editors, reviewers, and publishers in the academic community as a whole to be aware of these factors and strive for transparent reporting and consideration of all results, whether with high or low reliability, to maintain the credibility of L2 listening research.

The presence of publication bias in L2 listening test studies suggests that some researchers may overlook the role of reliability in their research. They may omit the reliability estimates if the statistical results are low. During the selection of research papers on L2 listening tests, a considerable number of studies were excluded because they did not report reliability coefficients. Specifically, 168 out of 260 journal articles were not included in the analysis due to this reason. Furthermore, a notable concern in L2 research is the insufficient interpretation of reliability, as noted by Plonsky and Derrick [4]. There remains a need to address this gap by providing L2 researchers and educators with guidelines on instrument reliability. To enhance the transparency and comprehensiveness of L2 listening research and mitigate publication bias, researchers should provide readers with detailed statistical results and thorough interpretations in their reports. This approach can contribute to a better understanding and offer valuable guidance regarding instrument reliability of L2 listening tests for both L2 investigators and educators.

Finally, it is important to note that low reliability in measurement does not necessarily signal a flaw in the test items or the underlying construct. For example, in scenarios where sample sizes are limited, the instrument may not yield highly reliable estimates due to the statistical limitation of small datasets [85] (p. 783). This is a reflection of the sample’s adequacy rather than the quality of the test itself. In addition, in the dynamic setting of a longitudinal study, what presents as low reliability might actually be due to positive changes among the test-takers, such as learning or development over time [85]. Here, the initial alignment of test items with the test-takers’ abilities can be disturbed as the participants’ proficiency improves over time, leading to what might be mistakenly interpreted as a decline in reliability. Therefore, rather than viewing low reliability as a definitive sign of problematic tools, it could be recognized as an indicator of development or a cue that the instrument may need recalibration to accommodate the test-takers’ advancements. Such insights underscore the importance of *contextual* and *temporal* factors in interpreting reliability, advocating for a dynamic approach to the application and continual adaptation of measurement instruments in language and listening assessments. In sum, studies with low reliability should not remove the reliability coefficient. In addition, journal reviewers and editors ought to take into account the context and unique features of studies before concluding that low reliability compromises a study’s validity.

### 6.2. Research Question 2: Meta-Regression Analysis of L2 Listening Tests

Heterogeneity, another key concept in RG meta-analysis, pertains to the variability observed in research outcomes [68]. Notably, the studies published on L2 listening tests exhibited considerable heterogeneity, signifying that various factors such as study contexts, participants’ demographics, and test characteristics might influence the reliability of L2 listening assessments.

To explore potential sources of heterogeneity in L2 listening test research, we conducted a meta-regression analysis in this study. All three categories from previous L2 listening tests, study contexts, participants’ demographics, and test features, were analyzed to determine the significant variables that affect the reliability of L2 listening tests.

*Study Context, Participant Demographics, and Test Features.* Although researchers commonly prefer involving 51–100 participants in L2 listening tests, the study results reveal that sample size does not significantly affect the reliability of L2 listening assessments. The finding is consistent with Aryadoust et al.’s [63] RG meta-analysis of LLM in L2 research which showed that the overall reliability of LLM instruments is not moderated by sample size. There is also no correlation between sample size and instrument reliability, interrater reliability, or intra-rater reliability in L2 research [4]. Given the consistency of these findings with other studies, it becomes essential to rectify any misconceptions that associate higher participant numbers with necessarily higher L2 language assessment reliability. In reality, the reliability of L2 listening tests cannot be defined solely based on the number of test-takers involved.

In contrast to the conclusions drawn by Plonsky and Derrick [4], the results of this study’s meta-regression analysis indicated that neither participants’ educational background nor their gender exerted a significant influence on the reliability of L2 listening tests. Plonsky and Derrick [4] posited that reliability within L2 research may be affected positively or negatively due to variations among participants, particularly when they are part of a homogenous group in terms of proficiency. It is noteworthy that the present study did not uncover a relationship between instrument reliability and participants’ L2 language proficiency levels, as Plonsky and Derrick [4] found. This might be due to the different classifications of participants in the two studies. Plonsky and Derrick [4] defined participants’ educational levels as low, intermediate, advanced, mixed, and native speakers. In contrast, the current study categorized participants (i.e., primary school students, middle school students, high school students, and university students) based on the information provided by the L2 listening test researchers. These contrasting classifications could potentially contribute to the dissimilar findings regarding the impact of participants’ proficiency levels on instrument reliability.

Additionally, it is noteworthy that most L2 listening studies have assessed the listening abilities of both male and female participants, yet they frequently overlook the potential impact of gender on language learning. According to Trang [86], gender-related differences in listening may exist between male and female students due to variations in brain hemisphere dominance during auditory processing. Whether such gender differences could indeed influence the reliability of L2 listening tests remains a topic that needs future research. A more comprehensive exploration, involving a larger number of studies, is required to fully understand the potential role of gender in affecting the reliability of L2 listening assessments.

Test features are considered a major factor affecting the reliability of language assessment [9]. In this study, three types of test features were collected to investigate their impact on the reliability of L2 listening tests: test type, the number of items, and item type. Surprisingly, item type was found to be statistically non-significant in terms of their effect on the reliability of L2 listening tests. This contradicts previous research, as other studies have indicated that item type could influence the reliability of language assessment and L2 research [55]. For instance, multiple-choice questions (MCQs) are viewed as a highly reliable test form in language assessment [48]. However, this study found no relationship between item type (MCQs, constructed-response items, and mixed method) and the reliability of L2 listening tests. In addition, lab-based research has higher instrument reliability than classroom-based studies in L2 research as researchers can have better experimental control in the lab [4]. One potential explanation for these discrepancies may be that a number of previous L2 listening studies have not provided comprehensive information about test features like test form and test setting. Consequently, the relatively limited sample size for these three variables in the meta-regression analysis could have contributed to divergent findings compared to earlier research. Notably, the number of items and test type were identified as key factors affecting the reliability of L2 listening tests, and these factors will be discussed further in the following sections.

*The Number of Items.* In contrast to sample size, the number of items is a significant factor influencing the reliability of L2 listening tests. It can be interpreted from the data that L2 listening tests with more test items yield higher reliability than L2 listening tests with fewer test items. This observation is consistent with findings in language assessment, where test length, or the number of items, has been shown to affect test reliability. Specifically, longer language tests tend to exhibit higher reliability [48]. In addition, other studies also find the importance of the number of items in L2 cloze tests [62] and LLM in L2 research [63]. This consistent pattern emphasizes the role of test length and item quantity in influencing the reliability of L2 assessments, highlighting the importance of carefully considering the number of items when designing L2 listening tests.

The preference among most researchers to include over 30 test items in their L2 listening tests may be attributed to the nature of these tests, which serve not only an academic purpose but also aim to assess L2 learners’ listening abilities in real-life contexts [87]. Thus, the number of items in the test is increased by using different types of listening materials in an L2 listening test to assess learners’ listening ability from various perspectives. Some large-scale standardized listening tests examine both L2 learners’ listening comprehension and interaction between test takers and tests. For instance, the listening section of IELTS contains 40 test items with four different listening materials such as conversations, news reports, and monologues to assess not only listeners’ listening but also their reading and writing. Despite this, the present RG meta-analysis did not gather specific details about the listening materials employed in the L2 listening tests under study. This limitation underscores the need for additional research to investigate the potential relationship between the reliability of L2 listening tests and the quantity and diversity of listening materials utilized. A deeper investigation with ample information on the listening materials could shed light on how test items and materials collectively impact the reliability of L2 listening assessments.

Furthermore, the reliability of L2 listening tests can indeed be enhanced by incorporating a greater number of test items. This is in line with the algebraic foundations of the coefficient where k/k−1 is used as a correction factor to adjust for the effect of items especially when the test consists of only a few items. Including more construct-relevant test items or tasks amplifies the diversification of the tasks and prevents undue influence from being a small pool of topics and test items [1]. This expansion can further increase the sample of behavior and augment the stability of the measurement, thus reducing error. Empirical studies corroborate these postulations. For example, Livingston et al. [48] demonstrated that the reliability of a test is increased by adding the test items and decreased by reducing test items in a test. This insight underscores the significance of considering test length or item quantity when evaluating L2 learners’ listening abilities.

When evaluating L2 learners’ listening ability, researchers and educators should consider including more test items in listening tests to enhance their internal consistency. A practical example can be found in Liu et al.’s [47] research, where they effectively improved the reliability of their English listening test by expanding its scale with the inclusion of more test items. Thus, L2 listening tests featuring an increased number of test items can offer a comprehensive examination of learners’ listening abilities from diverse angles. This approach may be effective in differentiating between high-level and low-level students based on the reliable scores obtained from L2 listening tests.

*Test Type.* L2 listening tests typically fall into two common categories: standardized tests and researcher-designed or teacher-designed tests. Standardized L2 listening tests refer to commercialized L2 listening tests developed by official organizations. A significant number of L2 learners have to opt for these tests to evaluate their L2 listening proficiency. On the contrary, researcher-designed or teacher-designed L2 listening tests are created by individuals for specific research or examination purposes. These tests may be administrated on a smaller scale with a shorter length compared to standardized tests.

According to the result of the meta-regression analysis, test type emerges as another influential factor that can impact the reliability of L2 listening tests. That is, standardized L2 listening tests may yield higher reliability than researcher-designed or teacher-designed L2 listening tests. Chiedu and Omenogor [9] also found the role of test type in the reliability of language assessment. Small-scale language tests designed by teachers and used in classrooms or schools May yield comparatively reduced reliability coefficients than standardized tests.

Several reasons might contribute to this finding. On the one hand, the development process of standardized L2 listening tests tends to be more intricate compared to researcher-designed or teacher-designed tests. Official organizations often afford to engage a number of expert designers and a team of professional L2 listening test developers during the selection of L2 listening materials. Conversely, researcher-designed or teacher-designed tests are usually crafted by the researchers or teachers themselves for their specific participants or students. Furthermore, standardized L2 listening tests supposedly undergo pilot testing to ensure their reliability and validity, whereas this step is often limited in researcher-designed or teacher-designed tests due to time and resource constraints.

It is important to note that the content and objectives of standardized L2 listening tests differ from those of researcher-designed or teacher-designed tests. Standardized tests assess general L2 learners’ listening ability, while the latter focus on specific groups of participants or students based on their study goals and teaching objectives, and as such, they may be criterion-referenced rather than norm-referenced. The RG analysis results could suggest that norm-referenced L2 listening tests, tailored to broader populations with an emphasis on minimizing measurement errors, are more amenable to reliability analysis. In contrast, criterion-referenced listening tests, being more specialized and context-dependent, may exhibit different reliability dynamics and may not always be conducive to conventional reliability evaluations. These postulations can be submitted for further exploration in future research.

### 6.3. Research Question 3: The Relationship between L2 Listening Test Scores and Constructs

Previous research has found that linguistic factors (e.g., vocabulary), cognitive factors (e.g., working memory), and affective factors (e.g., anxiety) impact L2 listening [88]. Nevertheless, the moderating effects of reliability analysis on these relationships have never been examined previously. Although the mean reliability of studies was high, it was found that the overall correlation coefficients of L2 listening tests including studies of impacts of vocabulary, listening strategy instruction, metacognitive awareness, and working memory on L2 listening are not moderated by the reliability of L2 listening tests. This finding, even though derived from a small sample, has implications for validity research.

Validity argument frameworks consider validity as a multifaceted argument in which reliability or generalization is a prerequisite for a higher-level inference such as the explanation or extrapolation inference [59]. It may be said that the correlation between L2 listening test scores and pertinent cognitive tests such as linguistic factors and working memory provides support for construct validity for the explanation inference of these tests. Accordingly, it may be posited that those correlations, which represent evidence for construct solidity, should be partially contingent upon evidence for reliability or lower-level inference in the validity argument framework [7]. However, this was not found in this study. We may cautiously suggest that reliability may not necessarily be a facet of validity in listening assessment.

One possible reason could be the small sample size within each category of the study. There were only one to two studies investigating metacognition, working memory, and strategy instruction, which failed to show a significant relationship between the average reliability and correlations. In addition, the small size of 12 studies on vocabulary and listening is still too small to allow for a rigorous correlational study. Bland [89] and Jenkins and Quintana-Ascencio [90] recommend minimum sample sizes of 19 and 25, respectively, for attaining a medium-sized coefficient in research, where there is a true correlation among variables. In addition, Algina and Olejnik [91] have presented tables detailing the required sample sizes for correlation analysis, aimed at achieving specific levels of research precision. The lack of correlation observed in our study may also be attributed to the redundancy of test items, especially in standardized listening tests, a characteristic noted in 14 out of the 17 studies included in our correlation analysis. As highlighted by Nimon et al. [92], redundant items may increase reliability without enhancing test validity, as an excessive number of test items can result in high item interrelations which might not accurately reflect actual language proficiency, as suggested by Boyle [93]. Despite the absence of correlations observed, the mean correlation coefficient between vocabulary and L2 listening test scores (0.57) was consistent with Zhang and Zhang’s [64] finding that indicated the “true” coefficient between L2 vocabulary and listening test scores may range between 0.56 and 0.67.

Overall, due to the small sample size in this study, further investigation is needed to determine whether the reliability of L2 listening tests affects other statistical results within L2 listening research. To our knowledge, this study would be the first investigation of the effect of reliability on the relationship between relevant constructs. This potential avenue of research holds the promise of providing researchers and educators with a deeper understanding and interpretation of various statistical aspects within the realm of L2 listening studies. We recommend that with a larger sample, advanced predictive modeling techniques such as regression or path analysis be used to explore the potential impact of reliability on the nomological relationships between relevant constructs.

### 6.4. Research Question 4: Reliability Induction

Reliability induction relies on past studies and may not accurately represent the reliability of present research. The result of the Mann–Whitney U test suggested that reliability induction from L2 listening studies indeed exerts a significant influence on the reliability of L2 listening tests. Previous studies also suggested that reliability induction played an important role in reporting reliability. Vassar et al. [70] found that a large number of life satisfaction studies induced reliability from previous reports but none of them provided valid justification for extracting reliability.

Reliability coefficients were yielded in terms of previous studies which are different from researchers’ present studies. For instance, several researchers referenced the reliability of IELTS from previous research to prove the internal consistency of the listening section of IELTS in their studies. However, the IELTS consists of four sections: listening, reading, writing, and speaking. The reliability of the whole test could not represent the reliability of the listening section. Even if the reliability of the listening section is reported in previous studies, researchers still cannot ensure the reliability of their study as there are numerous IELTS tests and the tests used may not be the same as tests used in previous research. Given that test features significantly affect the reliability of L2 listening tests, it is crucial to assess the reliability of these tests according to the parameters of the current study rather than relying solely on past research findings. Reliability induction serves as a reference point, offering researchers a preliminary understanding of the assessment before delving into an in-depth examination of the reliability of L2 listening tests. According to Vacha-Haase et al. [69], referencing reliability from previous research is preferable to not mentioning it at all, as it shows that researchers recognize the importance of reliability. However, not reporting the reliability coefficients of current studies prevents both researchers and readers from fully understanding the results.

## 7. Future Research

There is a great need to conduct future studies in the area of reliability in L2 listening assessment. Subsequent research should (1) expand the present study’s scope by incorporating a broader range of L2 listening test studies, including multiple languages and databases; (2) involve a more extensive sample size to confirm the reproducibility of the current results, given that the sample size for moderator analysis and reliability induction analysis in this study was comparatively limited; and (3) offer a detailed account of the research methods and procedures to enhance other researchers’ understanding of their prospective studies.

In addition, Cronbach’s alpha has several notable weaknesses, which warrant the use of alternative reliability measures. Cronbach’s alpha assumes the unidimensionality of the test items [94], meaning that it presumes all test items measure the same underlying construct, which is often not the case in complex scales. Another potential weakness is that the alpha coefficient implies equal weighting of all test items [95], disregarding their individual contributions or difficulties. Additionally, as the meta-regression analysis in this study has shown, the value of Cronbach’s alpha tends to inflate with the increase in the number of test items, which can mislead interpretations about the test’s reliability. The assumption of tau-equivalence is another limitation, where it is presumed each item has the same true score variance [96]. The potential for misinterpretation of its values [97] and its insensitivity to item-order effects [98] further limit its applicability in diverse research contexts.

As previously discussed, there are other types of reliability measures beyond Cronbach’s alpha coefficient that have yet to be utilized in L2 listening assessments. We specifically encourage future researchers to consider test–retest reliability and parallel-form reliability analysis [1]. The advantage of these two types of reliability measures over Cronbach’s alpha is that they provide a more comprehensive understanding of the stability and consistency of the assessment tool. Test–retest reliability assesses the temporal stability of the test, ensuring that the assessment yields consistent scores over time. This is important for determining whether changes in test scores are sensitive to changes in the listening construct being measured, rather than fluctuations in the test’s precision. Parallel-forms reliability, on the other hand, evaluates the equivalence of different versions of a test, ensuring that each form measures the same construct with the same level of accuracy. This is particularly important in scenarios where multiple test forms are used to avoid cheating or repetition effect (see [40] for an example of its application in listening assessment).

## 8. Conclusions

In summary, the present study found that the overall reliability of L2 listening tests was high. However, there was evidence of heterogeneity and potential publication bias in the published journal articles. Two key factors affecting the reliability of L2 listening tests were identified: the number of items and test type. Tests with more items and standardized formats generally exhibited higher reliability. Additionally, the relationship between L2 listening test scores and other constructs was not associated with the reliability coefficients of these tests. Furthermore, the reliability induction of L2 listening test scores impacted their internal consistency.

Many researchers did not report reliability estimates in their L2 listening studies, reflecting a lack of emphasis on the role of reliability in L2 listening tests. The absence of these estimates can threaten the replicability of L2 listening research, increase the margin of error in test scores, and potentially undermine the study’s validity. In the presence of such high measurement errors, the results of statistical analysis are affected by type I and II errors [99]. Readers of such reports would also have no idea about L2 listening tests (instruments) researchers used in L2 listening studies. Thus, it is necessary for researchers to provide sufficient information about L2 listening tests and other instruments they adopted in the research. Moreover, although reliability coefficients of some L2 listening tests are reported, they are seldom interpreted in the research. Correct interpretations of the reliability of L2 listening tests allow both researchers and readers to better understand the precision of these tests and the results of related studies. It is essential to enhance researchers’ awareness of L2 listening test reliability and provide training on how to interpret these reliability measures effectively.

Findings from this study also have implications for teachers in L2 listening assessment. On the one hand, L2 teachers need to gain knowledge about the reliability of L2 listening tests [1]. When assessing students’ L2 listening ability, teachers should be aware of the reliability of L2 listening tests as reliable L2 listening tests can assess listeners’ listening more precisely than unreliable tests. On the contrary, tests that tend to yield unreliable scores may lead to the misinterpretation of L2 learners’ listening ability. Conversely, it is also crucial for teachers to consider factors that influence the reliability of L2 listening test scores, such as the number of relevant items and test type. Teachers can enhance the reliability of L2 listening tests by increasing the number of relevant test items in their criterion-referenced assessments.

## Figures and Tables

**Table 1:** Inclusion and exclusion criteria.

Inclusion Criteria (Papers...)	Exclusion Criteria (Papers...)
were published in a peer-reviewed journal. were presented in English. included L2 listening tests. investigated and reported the reliability of L2 listening tests.	are not peer-reviewed journal articles. are not presented in English. do not include L2 listening tests. do not report the reliability of L2 listening tests.

**Table 2:** Coding scheme categories, variables, and definitions.

Variable	Definition
*Study Descriptions*	
NO.	The order of the study
Author	Author of the study
Year	Year publication
Country	The country of the study
Sample size	Number of participants
L2 language	Participants’ second language
L1 language Background *Test Features* Test type Item type Test setting Test form Test time Number of listening materials *Statistical Results* ^2^ Number of items Reliability coefficient	Participants’ first language Educational level of participants: primary school students, middle school students, high school students, university students, cram school students, adult learners, EFL learners or test takers ^1^ Type of the test items in an L2 listening test: standardized test or researcher-designed test Method of test in an L2 listening test: MCQs constructed-response items, or mixed The place of the L2 listening test taken place: classroom or lab The form of the test: paper-based or computer-based test Time spent to finish the L2 listening test The quantity of listening materials in an L2 listening test Quantity of items in an L2 listening test The reliability coefficient of an L2 listening test (type of measure and value)

Notes: ^1^ The background categories used in our analysis were directly sourced from the studies themselves, without any interpretation on our part. ^2^ The length and number of listening passages were not coded due to unavailability and inconsistency of data across the studies included.

**Table 3:** Summary of participant demographics.

Category	*k*	*%*	Mean Reliability of L2 Listening Tests	Standard Deviation
*Background*				
Primary school students	4	3.20	0.81	0.012
Middle school students	6	4.90	0.84	0.079
High school students	9	7.30	0.75	0.085
University students	68	55.70	0.82	0.088
Adult learners	10	8.10	0.76	0.098
EFL learners/ESL learners	18	14.70	0.83	0.103
Test takers	4	3.20	0.83	0.087
Cram school students	2	1.60	0.72	0.014
Not reported (missing)	1	1.30		
*Gender*				
Male and female	113	92.70	0.81	0.090
Female only	9	7.30	0.80	0.102
*L1 Language*				
Arabic	3	2.40	0.86	0.023
Chinese	37	30.30	0.81	0.082
Farsi	5	4.10	0.74	0.077
Japanese	18	14.80	0.80	0.114
Korea	7	5.70	0.83	0.075
Persian	6	4.90	0.81	0.091
Thai	2	1.60	0.80	0.078
Vietnamese	2	1.60	0.86	0.081
More than one language	38	31.10	0.79	0.081
*L2 Language*				
English	122	100	0.81	0.091

**Table 4:** Summary of study contexts.

Category	*k*	*%*	Mean Reliability of L2 Listening Tests	Standard Deviation
*Study’s Country*				
Australia	4	3.20	0.87	0.018
China	37	30.30	0.81	0.082
Iran	18	14.80	0.78	0.085
Japan	18	14.80	0.80	0.114
Korea	9	7.30	0.84	0.067
New Zealand	2	1.60	0.89	0.014
Saudi Arabia	3	2.40	0.86	0.023
Thailand	2	1.60	0.80	0.078
United States	13	10.70	0.76	0.103
Vietnam	2	1.60	0.86	0.080
Not reported (missing)	14	11.50		
*Sample Size*				
<50	11	9.00	0.83	0.096
51–100	39	32.00	0.79	0.084
101–150	28	23.00	0.82	0.101
151–200	8	6.60	0.83	0.082
201–250	10	8.20	0.82	0.089
251–300	3	2.50	0.81	0.035
More than 300	23	18.90	0.78	0.099

**Table 5:** Summary of test features.

Category	*k*	%	Mean Reliability	Standard Deviation
*Test type*				
Standardized tests	65	53.20	0.82	0.083
Researcher-designed tests	56	46.00	0.79	0.095
Not reported (missing)	1	0.80		
*Item type*				
MCQ	53	43.40	0.80	0.095
Item	54	44.30	0.81	0.092
Mixed	15	12.30	0.83	0.068
*Test setting*				
Classroom	34	27.90	0.79	0.092
Lab	7	5.70	0.82	0.062
Not reported (missing)	81	66.40		
*Test form*				
Paper-based	25	20.50	0.79	0.090
Computer-based	5	4.10	0.81	0.060
Not reported (missing)	92	75.40		

Note: The standardized tests found are commercialized L2 listening tests developed by test development organizations and companies, such as TOEFL and IELTS.

**Table 6:** Descriptive statistics of the number of items.

Category	Range	Minimum	Maximum	Mean	Standard Deviation
The number of items	208	8	216	40.93	31.996

**Table 7:** Coefficients of the meta-regression analysis.

Variable	Estimate	Std. Error	*t* Value	*p* Value
(Intercept)	0.662	0.070	9.352	0.000
The number of items	0.001	0.000	4.185	0.000
Test Type	0.037	0.016	2.350	0.021
Item Type (MCQ) Item Type (mixed)	0.001 0.008	0.018 0.025	0.077 0.316	0.938 0.753
Background (EFL learners)	0.040	0.034	1.184	0.239
Background (High school students)	−0.024	0.040	−0.602	0.549
Background (Cram school students)	−0.080	0.065	−1.233	0.220
Background (Middle school students)	0.067	0.044	1.522	0.131
Background (Primary school students)	0.051	0.049	1.029	0.306
Background (Test takers)	0.025	0.056	0.450	0.653
Background (University students)	0.027	0.029	0.915	0.362
Sample Size	0.000	0.000	0.924	0.358
Gender	−0.009	0.030	−0.285	0.776

**Table 8:** Descriptive statistics of correlation studies.

Category	k	%	Mean Correlation between Listening Score and the Construct	Mean Reliability	Correlation between Listening Test Reliability and Construct Correlations with Listening
Vocabulary	12	9.80	0.57	0.84	0.098
Metacognitive awareness	2	1.60	0.46	0.87	0.328
Working memory	2	1.60	0.62	0.85	
Strategy instruction	1	.80	0.44	0.80	

**Table 9:** Descriptive statistics of reliability studies.

Category	k	%	Mean Reliability in L2 Listening Studies	Standard Deviation
Reliability induction	5	4.10	0.89	0.263
Other studies	117	95.90	0.80	0.906

## Data Availability

No new data were created or analyzed in this study.

## Supplementary Materials

The following supporting information can be downloaded at: https://www.mdpi.com/article/10.3390/brainsci14080746/s1. Supplemental Material S1: Scopus search code; Supplemental Material S2: List of journals; Supplemental Material S3; List of publications; Supplemental Material S4: The (partial) R code for RG analysis.
