# Supplementary material for: A Meta-Analysis of the Reliability of Second Language Listening Tests (1991–2022)
Source: Brain Sci. 2024 Jul 25;14(8):746. doi: 10.3390/brainsci14080746 (PMC11353186; doi:10.3390/brainsci14080746)
Supplement: Supplementary file 1 [file brainsci-14-00746-s001.zip › brainsci-3093148-supplementary.pdf]

Supplemental materials

Supplemental material 1 Scopus search code

ALL (applied AND linguistics) OR (SRCTITLE ("applied linguistics") OR SRCTITLE ("modern language journal") OR SRCTITLE ("language learning") OR SRCTITLE ("language testing") OR SRCTITLE ("studies in second language acquisition") OR SRCTITLE ("journal of second language writing") OR SRCTITLE ("language teaching") OR SRCTITLE ("tesol quarterly") OR SRCTITLE ("language teaching research") OR SRCTITLE ("computer assisted language learning") OR SRCTITLE ("language learning and technology") OR SRCTITLE ("international journal of multilingualism") OR SRCTITLE ("bilingualism") OR SRCTITLE ("system") OR SRCTITLE ("assessing writing") OR SRCTITLE ("second language research") OR SRCTITLE ("studies in second language learning and teaching") OR SRCTITLE ("itl - international journal of applied linguistics") OR SRCTITLE ("international journal of bilingual education and bilingualism") OR SRCTITLE ("foreign language annals") OR SRCTITLE ("international multilingual research journal") OR SRCTITLE ("english for specific purposes") OR SRCTITLE ("research in the teaching of english") OR SRCTITLE ("journal of english for academic purposes") OR SRCTITLE ("reading and writing") OR SRCTITLE ("language awareness") OR SRCTITLE ("recall") OR SRCTITLE ("calico journal") OR SRCTITLE ("journal of multilingual and multicultural development") OR SRCTITLE ("international journal of bilingualism") OR SRCTITLE ("bilingual research journal") OR SRCTITLE ("language assessment quarterly") OR SRCTITLE ("elt journal") OR SRCTITLE ("language learning journal") OR SRCTITLE ("relc journal") OR SRCTITLE ("international journal of applied linguistics") OR SRCTITLE ("linguistic approaches to bilingualism") OR SRCTITLE ("aila review") OR SRCTITLE ("language acquisition") OR SRCTITLE ("applied linguistics review") OR SRCTITLE ("innovation in language learning and teaching") OR SRCTITLE ("reading and writing quarterly") OR SRCTITLE ("language testing in asia") OR SRCTITLE ("canadian modern language review") OR SRCTITLE ("tesol journal") OR SRCTITLE ("tesol international journal") OR SRCTITLE ("study abroad research in second language acquisition and international education") OR SRCTITLE ("english teaching") OR SRCTITLE ("teaching english with technology") OR SRCTITLE ("chinese journal of applied linguistics") OR SRCTITLE ("jalt call journal") OR SRCTITLE ("journal of asia tefl") OR SRCTITLE ("iranian journal of language teaching research") OR SRCTITLE ("asian esp journal") OR SRCTITLE ("iral - international review of applied linguistics in language teaching")) AND (LIMIT-TO (EXACTSRCTITLE , "System") OR LIMIT-TO (EXACTSRCTITLE , "Iral International Review Of Applied Linguistics In Language Teaching") OR LIMIT-TO (EXACTSRCTITLE , "Applied Linguistics") OR LIMIT-TO (EXACTSRCTITLE , "Tesol Quarterly") OR LIMIT-TO (EXACTSRCTITLE , "Language Learning") OR LIMIT-TO (EXACTSRCTITLE , "Modern Language Journal") OR LIMIT-TO (EXACTSRCTITLE , "Journal Of Multilingual And Multicultural Development") OR LIMIT-TO (EXACTSRCTITLE , "Language Teaching Research") OR LIMIT-TO (EXACTSRCTITLE , "Studies In Second Language Acquisition") OR LIMIT-TO (EXACTSRCTITLE , "International Journal Of Bilingual Education And Bilingualism") OR LIMIT-TO (EXACTSRCTITLE , "Foreign Language Annals") OR LIMIT-TO (EXACTSRCTITLE , "English For Specific Purposes") OR LIMIT-TO (EXACTSRCTITLE , "Bilingualism") OR LIMIT-TO (EXACTSRCTITLE , "Journal Of

English For Academic Purposes") OR LIMIT-TO (EXACTSRCTITLE , "Language Teaching") OR LIMIT-TO (EXACTSRCTITLE , "Relc Journal") OR LIMIT-TO (EXACTSRCTITLE , "Second Language Research") OR LIMIT-TO (EXACTSRCTITLE , "International Journal Of Bilingualism") OR LIMIT-TO (EXACTSRCTITLE , "Language Learning Journal") OR LIMIT-TO (EXACTSRCTITLE , "Computer Assisted Language Learning") OR LIMIT-TO (EXACTSRCTITLE , "Language Testing") OR LIMIT-TO (EXACTSRCTITLE , "Journal Of Asia Tefl") OR LIMIT-TO (EXACTSRCTITLE , "Language Awareness") OR LIMIT-TO (EXACTSRCTITLE , "Journal Of Second Language Writing") OR LIMIT-TO (EXACTSRCTITLE , "Canadian Modern Language Review") OR LIMIT-TO (EXACTSRCTITLE , "International Journal Of Multilingualism") OR LIMIT-TO (EXACTSRCTITLE , "Elt Journal") OR LIMIT-TO (EXACTSRCTITLE , "Reading And Writing") OR LIMIT-TO (EXACTSRCTITLE , "Applied Linguistics Review") OR LIMIT-TO (EXACTSRCTITLE , "Language Learning And Technology") OR LIMIT-TO (EXACTSRCTITLE , "Calico Journal") OR LIMIT-TO (EXACTSRCTITLE , "Recall") OR LIMIT-TO (EXACTSRCTITLE , "Language Assessment Quarterly") OR LIMIT-TO (EXACTSRCTITLE , "International Journal Of Applied Linguistics") OR LIMIT-TO (EXACTSRCTITLE , "Innovation In Language Learning And Teaching") OR LIMIT-TO (EXACTSRCTITLE , "Tesol Journal") OR LIMIT-TO (EXACTSRCTITLE , "Asian Esp Journal") OR LIMIT-TO (EXACTSRCTITLE , "Linguistic Approaches To Bilingualism") OR LIMIT-TO (EXACTSRCTITLE , "Assessing Writing") OR LIMIT-TO (EXACTSRCTITLE , "Bilingual Research Journal") OR LIMIT-TO (EXACTSRCTITLE , "Chinese Journal Of Applied Linguistics") OR LIMIT-TO (EXACTSRCTITLE , "Studies In Second Language Learning And Teaching") OR LIMIT-TO (EXACTSRCTITLE , "Iranian Journal Of Language Teaching Research") OR LIMIT-TO (EXACTSRCTITLE , "Language Acquisition") OR LIMIT-TO (EXACTSRCTITLE , "International Multilingual Research Journal") OR LIMIT-TO (EXACTSRCTITLE , "Language Testing In Asia") OR LIMIT-TO (EXACTSRCTITLE , "Aila Review") OR LIMIT-TO (EXACTSRCTITLE , "Itl International Journal Of Applied Linguistics Belgium") OR LIMIT-TO (EXACTSRCTITLE , "English Teaching") OR LIMIT-TO (EXACTSRCTITLE , "Teaching English With Technology") OR LIMIT-TO (EXACTSRCTITLE , "Tesol International Journal") OR LIMIT-TO (EXACTSRCTITLE , "Reading And Writing Quarterly") OR LIMIT-TO (EXACTSRCTITLE , "Research In The Teaching Of English") OR LIMIT-TO (EXACTSRCTITLE , "Jalt Call Journal") OR LIMIT-TO (EXACTSRCTITLE , "Study Abroad Research In Second Language Acquisition And International Education"))

Supplemental material 2 List of journals

Applied Linguistics	2
Asian-Pacific of Second and Foreign Language Education	1
Canadian Journal of Applied Linguistics	1
Cogent Education	1
Frontiers in Psychology	2
Innovation in Language Learning and Teaching	2
ITL - International Journal of Applied Linguistics (Belgium)	1

International Journal of Bilingualism	1
International Journal of Instruction	1
Iranian Journal of Language Teaching Research	1
Journal of Asia TEFL	3
Journal of Computer Assisted Learning	1
Journal of English for Academic Purposes	1
Journal of Language and Education	3
Journal of Psycholinguistic Research	1
Language Assessment Quarterly	6
Language Awareness	2
Language Learning	4
Language Learning and Technology	5
Language Teaching Research	5
Language Testing	13
Language Testing in Asia	4
Modern Journal of Language Teaching Methods	2
Modern Language Journal	1
RELC Journal	5
Studies in Second Language Acquisition	4
System	14
TESOL Quarterly	6

Supplemental material 3 List of publications

Alavi, S. M., & Janbaz, F. (2014). Comparing two pre-listening supports with iranian EFL learners: Opportunity or obstacle. *RELC Journal*, *45*(3), 253–267. https://doi.org/10.1177/0033688214546963

Amiryousefi, M. (2019). The incorporation of flipped learning into conventional classes to enhance EFL learners' L2 speaking, L2 listening, and engagement. *Innovation in Language Learning and Teaching*, *13*(2), 147–161. https://doi.org/10.1080/17501229.2017.1394307

Aryadoust, V., Goh, C. C. M., & Kim, L. O. (2011). An investigation of differential item functioning in the MELAB listening test. *Language Assessment Quarterly*, *8*(4), 361–385. https://doi.org/10.1080/15434303.2011.628632

Brunfaut, T., & Révész, A. (2015). The Role of Task and Listener Characteristics in Second Language Listening. *TESOL Quarterly*, *49*(1), 141–168. https://doi.org/10.1002/tesq.168

Buck, G., & Tatsuoka, K. (1998). Application of the rule-space procedure to language testing: Examining attributes of a free response listening test. *Language Testing*, *15*(2), 119–157. https://doi.org/10.1177/026553229801500201

Cai, H. (2013). Partial dictation as a measure of EFL listening proficiency: Evidence from confirmatory factor analysis. *Language Testing*, *30*(2), 177–199. https://doi.org/10.1177/0265532212456833

Cai, H. (2020). Relating Lexical and Syntactic Knowledge to Academic English Listening: The Importance of Construct Representation. *FRONTIERS IN PSYCHOLOGY*, *11*. https://doi.org/10.3389/fpsyg.2020.00494

Chang, A. C.-S., & Millett, S. (2016). Developing L2 Listening Fluency through Extended Listening-focused Activities in an Extensive Listening Programme. *RELC Journal*, *47*(3), 349–362. https://doi.org/10.1177/0033688216631175

Chang, A. C.-S., & Read, J. (2007). Support for foreign language listeners: Its effectiveness and limitations. *RELC Journal*, *38*(3), 375–394. https://doi.org/10.1177/0033688207085853

Chang, A. C.-S., & Read, J. (2013). Investigating the effects of multiple-choice listening test items in the oral versus written mode on L2 listeners' performance and perceptions. *System*, *41*(3), 575–586. https://doi.org/10.1016/j.system.2013.06.001

Chang, A., Millett, S., & Renandya, W. (2019). Developing Listening Fluency through Supported Extensive Listening Practice. *RELC JOURNAL*, *50*(3), 422–438. https://doi.org/10.1177/0033688217751468

Chen, L., Zhang, R., & Liu, C. (2014). Listening strategy use and influential factors in Web-based computer assisted language learning. *JOURNAL OF COMPUTER ASSISTED LEARNING*, *30*(3), 207–219. https://doi.org/10.1111/jcal.12041

Cheng, J., & Matthews, J. (2018). The relationship between three measures of L2 vocabulary knowledge and L2 listening and reading. *LANGUAGE TESTING*, *35*(1), 3–25. https://doi.org/10.1177/0265532216676851

Cheng, J., Matthews, J., Lange, K., & Mclean, S. (2023). Aural single-word and aural phrasal verb knowledge and their relationships to L2 listening comprehension. *TESOL QUARTERLY*, *57*(1), 213–241. https://doi.org/10.1002/tesq.3137

Dai, D. W., & Roever, C. (2019). Including L2-English Varieties in Listening Tests for Adolescent ESL Learners: L1 Effects and Learner Perceptions. *Language Assessment Quarterly*, *16*(1), 64–86. https://doi.org/10.1080/15434303.2019.1601198

Delvand, S., & Heidar, D. (2020). Computerized Group Dynamic Assessment and Listening Comprehension Ability: Does Self- Efficacy Matter? *JOURNAL OF LANGUAGE AND EDUCATION*, *6*(1), 157–172. https://doi.org/10.17323/jle.2020.9834

Fathi, J., & Hamidizadeh, R. (2019). The Contribution of Listening Strategy Instruction to Improving Second Language Listening Comprehension: A Case of Iranian EFL Learners. *INTERNATIONAL JOURNAL OF INSTRUCTION*, *12*(2), 17–32. https://doi.org/10.29333/iji.2019.1222a

Ghaemi, H. (2022). Application of nonparametric item response theory in determining the one-dimensionality and scalability of TOEFL iBT listening test. *Language Testing in Asia*, *12*(1). https://doi.org/10.1186/s40468-022-00171-8

Ghasemboland, F., & Nafissi, Z. (2012). *The Effects of Using English Captions on Iranian EFL Students' Listening Comprehension* (A. Isman, E. Liu, & M. Kiyici, Eds.; WOS:000317142200013; Vol. 64, pp. 105–112). https://doi.org/10.1016/j.sbspro.2012.11.013

Goh, C. C. M., & Hu, G. (2014). Exploring the relationship between metacognitive awareness and listening performance with questionnaire data. *Language Awareness*, *23*(3), 255–274. https://doi.org/10.1080/09658416.2013.769558

Gorjian, B., Hayati, A., Matori, H., Abolghasemzadeh, A., & Rezapour, G. (2012). *THE EFFECT OF SPEECH RATE ON LISTENING COMPREHENSION OF EFL LEARNERS* (L. Chova, I. Torres, & A. Martinez, Eds.; WOS:000326239307070; pp. 7467–7475).

Ha, H. T. (2021a). A Rasch-based validation of the Vietnamese version of the Listening Vocabulary Levels Test. *Language Testing in Asia*, *11*(1). https://doi.org/10.1186/s40468-021-00132-7

Ha, H. T. (2021b). Exploring the relationships between various dimensions of receptive vocabulary knowledge and L2 listening and reading comprehension. *Language Testing in Asia*, *11*(1). Scopus. https://doi.org/10.1186/s40468-021-00131-8

Harding, L. (2012). Accent, listening assessment and the potential for a shared-L1 advantage: A DIF perspective. *Language Testing*, *29*(2), 163–180. https://doi.org/10.1177/0265532211421161

Harding, L., Pill, J., & Ryan, K. (2011). Assessor decision making while marking a note-taking listening test: The case of the OET. *Language Assessment Quarterly*, *8*(2), 108–126. https://doi.org/10.1080/15434303.2011.556770

He, L., Xiong, L., & Min, S. (2022). Diagnosing listening and reading skills in the Chinese EFL context: Performance stability and variability across modalities and performance levels. *System*, *106*. https://doi.org/10.1016/j.system.2022.102787

Hidri, S. (2014). Developing and evaluating a dynamic assessment of listening comprehension in an EFL context. *Language Testing in Asia*, *4*(1). https://doi.org/10.1186/2229-0443-4-4

Hui, B., Wong, S., & Au, R. (2022). Reading aloud listening test items to young learners: Attention, item understanding, and test performance. *SYSTEM*, *108*. https://doi.org/10.1016/j.system.2022.102831

Kim, A. A., Tywoniw, R. L., & Chapman, M. (2022). Technology-Enhanced Items in Grades 1–12 English Language Proficiency Assessments. *Language Assessment Quarterly*, *19*(4), 343–367. https://doi.org/10.1080/15434303.2022.2039659

Kim, M., Nam, Y., & Crossley, S. (2022). Roles of working memory, syllogistic inferencing ability, and linguistic knowledge on second language listening comprehension for passages of different lengths. *LANGUAGE TESTING*, *39*(4), 593–617. https://doi.org/10.1177/02655322211060076

Koyama, D., Sun, A., & Ockey, G. J. (2016). The effects of item preview on video-based multiple-choice listening assessments. *Language Learning and Technology*, *20*(1), 148–165.

Li, C. (2019). Using a Listening Vocabulary Levels Test to Explore the Effect of Vocabulary Knowledge on GEPT Listening Comprehension Performance. *LANGUAGE ASSESSMENT QUARTERLY*, *16*(3), 328–344. https://doi.org/10.1080/15434303.2019.1648474

Li, Y., & Zhang, X. (2019). L2 Vocabulary Knowledge and L2 Listening Comprehension: A Structural Equation Model. *CANADIAN JOURNAL OF APPLIED LINGUISTICS*, *22*(1), 85–102.

Matthews, J., & Cheng, J. (2015). Recognition of high frequency words from speech as a predictor of L2 listening comprehension. *SYSTEM*, *52*, 1–13. https://doi.org/10.1016/j.system.2015.04.015

McNamara, T. F. (1991). Test dimensionality: IRT analysis of an ESP listening test1. *Language Testing*, *8*(2), 139–159. https://doi.org/10.1177/026553229100800204

Milliner, B., & Dimoski, B. (2021). The effects of a metacognitive intervention on lower-proficiency EFL learners' listening comprehension and listening self-efficacy. *LANGUAGE TEACHING RESEARCH*. https://doi.org/10.1177/13621688211004646

Mohsen, M., & Almudawis, S. (2021). Second Language Vocabulary Gains from Listening Versus Reading Comprehension Input: A Comparative Study. *JOURNAL OF PSYCHOLINGUISTIC RESEARCH*, *50*(3), 543–562. https://doi.org/10.1007/s10936-020-09690-y

Monteiro, K., & Kim, Y. (2020). The effect of input characteristics and individual differences on L2 comprehension of authentic and modified listening tasks. *System*, *94*. https://doi.org/10.1016/j.system.2020.102336

Nadri, M., Baghaei, P., & Zohoorian, Z. (2019). The contribution of cognitive abilities and general language proficiency to explaining listening comprehension in English as a foreign language. *COGENT EDUCATION*, *6*(1). https://doi.org/10.1080/2331186X.2019.1567010

Namaziandost, E., Hafezian, M., & Shafiee, S. (2018). Exploring the association among working memory, anxiety and Iranian EFL learners' listening comprehension. *ASIAN-PACIFIC JOURNAL OF SECOND AND FOREIGN LANGUAGE EDUCATION*, *3*(1). https://doi.org/10.1186/s40862-018-0061-3

Ockey, G. J., & French, R. (2016). From One to Multiple Accents on a Test of L2 Listening Comprehension. *Applied Linguistics*, *37*(5), 693–715. https://doi.org/10.1093/applin/amu060

O'Grady, S. (2022). Trialing alternative listening assessment tasks: Interactions between text authenticity, item focus and item presentation condition. *INNOVATION IN LANGUAGE LEARNING AND TEACHING*. https://doi.org/10.1080/17501229.2022.2109643

Patterson, A. (2021). Predicting second language listening functor comprehension probability with usage-based and embodiment approaches. *INTERNATIONAL JOURNAL OF BILINGUALISM*, *25*(3), 772–788. https://doi.org/10.1177/13670069211000851

Pearson, W. S. (2021). The predictive validity of the Academic IELTS test: A methodological synthesis. *ITL - International Journal of Applied Linguistics (Belgium)*, *172*(1), 85–120. https://doi.org/10.1075/itl.19021.pea

Read, J. (2002). The use of interactive input in EAP listening assessment. *Journal of English for Academic Purposes*, *1*(2), 105–119. https://doi.org/10.1016/S1475-1585(02)00018-8

Safa, M., & Beheshti, S. (2018). Interactionist and Interventionist Group Dynamic Assessment (GDA) and EFL Learners' Listening Comprehension Development. *IRANIAN JOURNAL OF LANGUAGE TEACHING RESEARCH*, *6*(3), 37–56.

Safa, M., & Motaghi, F. (2021). Cognitive vs. Metacognitive scaffolding strategies and EFL learners' listening comprehension development. *LANGUAGE TEACHING RESEARCH*. https://doi.org/10.1177/13621688211021821

Sakai, H. (2009). Effect of repetition of exposure and proficiency level in L2 listening tests. *TESOL Quarterly*, *43*(2), 360–372. https://doi.org/10.1002/j.1545-7249.2009.tb00179.x

Selamat, S., & Sidhu, G. (2013). *Enhancing Listening Comprehension: The role of Metacognitive Strategy Instruction (MetSI)* (I. Ismail, S. AbdRahman, N. Noordin, & S. Mustafa, Eds.; WOS:000347954100053; Vol. 90, pp. 421–430). https://doi.org/10.1016/j.sbspro.2013.07.111

Shin, S., Lee, S., & Lidster, R. (2021). Examining the effects of different English speech varieties on an L2 academic listening comprehension test at the item level. *LANGUAGE TESTING*, *38*(4), 580–601. https://doi.org/10.1177/0265532220985432

Sok, S., Shin, H., & Do, J. (2021). Exploring which test-taker characteristics predict young L2 learners' performance on listening and reading comprehension tests. *LANGUAGE TESTING*, *38*(3), 378–400. https://doi.org/10.1177/0265532221991134

Song, M. (2008). Do divisible subskills exist in second language (L2) comprehension? A structural equation modeling approach. *LANGUAGE TESTING*, *25*(4), 435–464. https://doi.org/10.1177/0265532208094272

Song, M.-Y. (2012). Note-taking quality and performance on an L2 academic listening test. *Language Testing*, *29*(1), 67–89. https://doi.org/10.1177/0265532211415379

Stæhr, L. S. (2009). Vocabulary knowledge and advanced listening comprehension in english as a foreign language. *Studies in Second Language Acquisition*, *31*(4), 577–607. https://doi.org/10.1017/S0272263109990039

Taguchi, N. (2011). The effect of L2 proficiency and study-abroad experience on pragmatic comprehension. *Language Learning*, *61*(3), 904–939. https://doi.org/10.1111/j.1467-9922.2011.00633.x

Takimoto, M. (2008). The effects of deductive and inductive instruction on the development of language learners' pragmatic competence. *Modern Language Journal*, *92*(3), 369–386. https://doi.org/10.1111/j.1540-4781.2008.00752.x

Takimoto, M. (2009). The effects of input-based tasks on the development of learners pragmatic proficiency. *Applied Linguistics*, *30*(1), 1–25. https://doi.org/10.1093/applin/amm049

Tobia, V., Ciancaleoni, M., & Bonifacci, P. (2017). Theoretical models of comprehension skills tested through a comprehension assessment battery for primary school children. *LANGUAGE TESTING*, *34*(2), 223–239. https://doi.org/10.1177/0265532215625705

Vafaee, P., & Suzuki, Y. (2020). The relative significance of syntactic knowledge and vocabulary knowledge in second language listening ability. *Studies in Second Language Acquisition*, *42*(2), 383–410. https://doi.org/10.1017/S0272263119000676

Wagner, E. (2010a). Test-takers' interaction with an L2 video listening test. *System*, *38*(2), 280–291. https://doi.org/10.1016/j.system.2010.01.003

Wagner, E. (2010b). The effect of the use of video texts on ESL listening test-taker performance. *Language Testing*, *27*(4), 493–513. https://doi.org/10.1177/0265532209355668

Wagner, E. (2013). An investigation of how the channel of input and access to test questions affect L2 listening test performance. *Language Assessment Quarterly*, *10*(2), 178–195. Scopus. https://doi.org/10.1080/15434303.2013.769552

Wallace, M. (2021). Exploring the Relationship Between L2 Listening and Metacognition After Controlling for Vocabulary Knowledge. *JOURNAL OF LANGUAGE AND EDUCATION*, *7*(3), 187–200. https://doi.org/10.17323/jle.2021.12685

Wallace, M., & Lee, K. (2020). Examining Second Language Listening, Vocabulary, and Executive Functioning. *FRONTIERS IN PSYCHOLOGY*, *11*. https://doi.org/10.3389/fpsyg.2020.01122

Wallace, M. P. (2022). Individual Differences in Second Language Listening: Examining the Role of Knowledge, Metacognitive Awareness, Memory, and Attention. *Language Learning*, *72*(1), 5–44. https://doi.org/10.1111/lang.12424

Wang, S.-Y., & Cha, K.-W. (2019). Foreign language listening anxiety factors affecting listening performance of Chinese EFL learners. *Journal of Asia TEFL*, *16*(1), 121–134. https://doi.org/10.18823/asiatefl.2019.16.1.8.121

Yanagawa, K. (2022). The role of bottom-up strategy instruction and proficiency level in L2 listening test performance: An intervention study. *Language Awareness*. https://doi.org/10.1080/09658416.2022.2161557

Zeleke, A. (2013). DOES VIEWING TEST ITEMS AT DIFFERENT TIMES MATTER IN ENGLISH FOR ACADEMIC PURPOSE LISTENING TEST? *MODERN JOURNAL OF LANGUAGE TEACHING METHODS*, *3*(2), 63–77.

Zhang, P. (2022). How does repetition affect vocabulary learning through listening to the teacher's explicit instruction? The moderating role of listening proficiency and preexisting vocabulary knowledge. *Language Teaching Research*. https://doi.org/10.1177/13621688221140521

Zhang, P., & Graham, S. (2020a). Learning Vocabulary Through Listening: The Role of Vocabulary Knowledge and Listening Proficiency. *Language Learning*, *70*(4), 1017–1053. https://doi.org/10.1111/lang.12411

Zhang, P., & Graham, S. (2020b). Vocabulary learning through listening: Comparing L2 explanations, teacher codeswitching, contrastive focus-on-form and incidental learning. *Language Teaching Research*, *24*(6), 765–784. https://doi.org/10.1177/1362168819829022

Supplemental material 4 The (partial) R Code for RG analysis

```
#ai=alpha;
#mi= the # of items/replications/parts of the measurement instrument
#ni= sample sizes
# Calculate effect sizes
dat <- escalc(measure="ARAW", ai=Reliability, mi=Items,
           ni=Size,
           slab=paste(Author, ", ", sep=""), data=MT)

# Calculate mean alpha
mean_alpha <- mean(df$alpha)

# bubble plot
p <- ggplot(df, aes(x = study, y = alpha, size = weight)) +
  geom_point(alpha = 0.5) + # Adjust alpha for bubble transparency
  geom_hline(yintercept = mean_alpha, linetype = "dashed", color = "red") + # Add dashed line for
mean alpha
  scale_size_continuous(range = c(3, 10)) + # Adjust the range for bubble sizes
  theme_minimal() + # Use a minimal theme for the plot
  labs(title = "Reliability Generalization Analysis for Cronbach's Alpha",
     x = "Study",
     y = "Alpha",
     size = "Weight") +
  theme(legend.position = "bottom") # Position the legend at the bottom

# print the plot
print(p)

# funnel plot
funnel(res)
funnel(res, ylim=c(0,.8), las=1)
funnel(res, ylim=c(0,.8), las=1, digits=list(1L,1))

# Ensure 'Background' and 'Itemtype' are factors
data2$background <- factor(data2$background)
data2$itemtype <- factor(data2$itemtype)

# Perform Meta-Regression
```

Please see:
https://bookdown.org/MathiasHarrer/Doing_Meta_Analysis_in_R/metareg.html
